# Depletion of microglia exacerbates injury and impairs function recovery after spinal cord injury in mice

**DOI:** 10.1038/s41419-020-2733-4

**Published:** 2020-07-13

**Authors:** Haitao Fu, Yanpeng Zhao, Die Hu, Song Wang, Tengbo Yu, Licheng Zhang

**Affiliations:** 1 Department of Orthopedics, Chinese PLA General Hospital, Beijing, China; 2 Department of Orthopaedics, The Affiliated Hospital of Qingdao University, Qingdao, China; 3 Qingdao Eye Hospital, Shandong Eye Institute, Shandong First Medical University & Shandong Academy of Medical Sciences, Qingdao, China; 4 School of Medicine, Nankai University, Tianjin, China

**Keywords:** Microglia, Spinal cord diseases

## Abstract

The role of microglia in spinal cord injury (SCI) remains ambiguous, partially due to the paucity of efficient methods to discriminate these resident microglia with blood-derived monocytes/macrophages. Here, we used pharmacological treatments to specifically eliminate microglia and subsequently to investigate the response of microglia after SCI in mice. We showed that treatment with colony stimulating factor 1 receptor (CSF1R) inhibitor PLX3397 eliminated ~90% microglia and did not affect other cell types in mouse spinal cord. PLX3397 treatment also induced a strong decrease in microglial proliferation induced by SCI. Depletion of microglia after SCI disrupted glial scar formation, enhanced immune cell infiltrates, reduced neuronal survival, delayed astrocyte repopulation, exacerbated axonal dieback, and impaired locomotor recovery. Therefore, our findings suggest microglia may play a protective role after SCI in mice.

## Introduction

Microglia are derived from primitive myeloid progenitors in the yolk sac during embryonic development^1^. As the main immune cells in central nervous system (CNS), microglia help to sculpt the mature CNS during development by removing apoptotic cells and inappropriate neural connections^2^. In the adult CNS, microglia constantly surveil the microenvironment for alterations resulting from injury or disease^2,3^. After sensing perturbations, microglia become activated, reorient their processes towards the lesion, and phagocytose cellular debris^4^.

However, the role of microglia in CNS injury remains controversial. Activated microglia release proinflammatory factors that cause neuronal death and contribute to the secondary tissue damage^2,5^. Inhibition of microglia proliferation reduces inflammatory response, alleviates neuronal death, and improves motor recovery after spinal cord injury (SCI)^6,7^. Conversely, several studies demonstrate beneficial roles of microglia in CNS injury. In a mouse model of stroke, microglia are proved to play a role in protecting neurons by regulating intracellular calcium levels^8^. In a mouse model of contusive SCI, microglia are identified as a key cellular component of the scar that develops after SCI to protect neural tissue^9^. In addition, one study show that microglia are irrelevant for neuronal degeneration and axon regeneration after acute crush injury of optic nerve^10^. Therefore, microglia may exert diverging roles depending on the context. Whether microglia are beneficial or detrimental for recovery after SCI remains unclear.

In addition to their potentially conflicting roles, the paucity of efficient methods to distinguish these resident microglia with blood-derived monocytes/ macrophages hamper the exploration of the specific roles of microglia after a CNS injury^11^. The newly developed pharmacologic strategies based on CSF1R inhibition specifically eliminate ~99% microglia in adult brain, whereas peripheral macrophages and other immune cells remained unaffected^10,12^. This method allows the study of the specific roles of microglia in CNS injury.

Here, we used PLX3397, a CSF1R inhibitor that specifically eliminate microglia to investigate the specific roles of microglia in spinal cord. We showed that PLX3397 treatment eliminated almost all microglia and the absence of microglia did not affect other cell types in spinal cord. Depletion of microglia in the context of SCI was associated with disorganized astroglial scar, reduced neuronal number, delayed astrocyte repopulation, aggravated axonal dieback, and reduced functional recovery. Therefore, microglia may have a beneficial effect on function recovery after SCI.

## Materials and methods

### Animal experiments

Female C57BL/6 mice (6-week-old) were used for all experiments. Mice were housed under controlled conditions with 12 h light/dark cycle and had free access to food and water at all time. All animal procedures were approved by the Institutional Animal Care and Use Committee of the Chinese PLA General Hospital and conducted in accordance with relevant guides of the Chinese Ministry of Public Health on the care and use of laboratory animals and in compliance with the ARRIVE (Animal Research: Reporting In Vivo Experiments) guidelines.

### Microglia depletion

To deplete microglia in vivo, mice were given the CSF1R inhibitor PLX3397 (pexidartinib; Selleckchem, Houston, TX, USA) formulated into AIN-76A standard diet at 290 mg/kg for 7 days^5^. AIN-76A standard diet was used as respective controls.

### Spinal cord injury

A full crush injury was performed similar to that previously described by Liu et al.^13^. Mice were anesthetized by intraperitoneal injections of sodium pentobarbital at 80 mg/kg. A midline incision was made over the thoracic vertebrae. Then, a laminectomy was conducted at the level of T10 segment until the spinal cord was exposed completely from side to side. The exposed spinal cord was then crushed for 2 s with modified forceps. The forceps were filed to a width of 0.1 mm for the last 4–5 mm of the tips. The spinal dura was intact after crushing. The muscle and skin were sutured. Post-operatively, mice were placed on a warming blanket until fully awake. Bladders were emptied manually twice daily to prevent urinary tract infections until mice were able to urinate independently.

### Automated capillary western blot (WES)

Mice were transcardially perfused with cold phosphate-buffered saline (PBS) and then the spinal cords were isolated from spinal columns for western blot analysis. Western blots were performed using WES, an automated capillary-based size sorting system (ProteinSimple, San Jose, CA, USA)^14^. All procedures were performed according to the manufacturer’s protocol. Briefly, 8 μL of diluted protein lysate was mixed with 2 μL of 5× fluorescent master mix and heated at 95 °C for 5 min. The samples, blocking reagent, wash buffer, primary antibodies, secondary antibodies, and chemiluminescent substrate were dispensed into designated wells in a microplate provided by the manufacturer. The plate was loaded into the instrument and protein was drawn into individual capillaries on a 25 capillary cassette provided by the manufacturer. Protein separation and immunodetection was performed automatically on the individual capillaries using default settings. The data was analyzed with Compass software (ProteinSimple). Primary antibodies used were anti-Iba1 antibody (abcam, ab153696), anti-βIII Tubulin antibody (abcam, ab78078), anti-GFAP antibody (abcam, ab4674), and anti-Olig2 antibody (abcam, ab109186).

### BDA tracing

This procedure is similar to what was described previously with modifications^13,15^. A midline incision was made over the skull to reveal bregma after mice were anesthetized. Then, a window in the skull was made with a microdrill to expose the sensorimotor cortex. To label corticospinal tract (CST) axons by anterograde tracing, four injections of 500 nl BDA (10%, Invitrogen, D1956) were injected into sensorimotor cortex at four sites through a glass pipette attached to a nanoliter injector at a rate of 1 nl/s. The four coordinates were as follows: 1.5 mm lateral, 0.6 mm deep, and 0.5 mm anterior; 0.0 mm, 0.5 mm, and 1.0 mm caudal to bregma. In order to prevent the backflow of the injection, the needle was kept in situ for 1 min before moving to the next site. The skin was sutured and mice were then placed on a warming blanket. These BDA-injected mice were kept for an additional 2 weeks before termination.

### Tissue processing

For the purpose of histology and immunofluorescence experiments, mice were anesthetized and transcardially perfused with cold PBS followed by 4% paraformaldehyde (PFA). Spinal columns were isolated and post-fixed in the 4% PFA overnight at 4 °C. A spinal cord segment of 10 mm containing the lesion site were dissected out and then immersed for 3 days at 4 °C in a PBS solution containing 30% sucrose for cryoprotection. Tissues were then embedded in OCT compound, snap-frozen in dry ice and stored at −20 °C until processed.

### HE and immunofluorescence stain

For HE stain, coronal sections of the spinal cord centered over the lesion site were cut on a cryostat at 25–30 μm and then stained using a commercial staining kit (Solarbio, G1120). For Immunofluorescence stain, coronal, sagittal, or transverse sections of the spinal cord were cut at 25–30 μm thickness. All steps of immunostaining were performed following standard protocols. Briefly, sections were rinsed with PBS for 5 min at room temperature, encircled with a hydrophobic barrier, and blocked for 2 h with 10% normal goat serum, 0.5% Triton X-100 in PBS. Primary antibodies used in this study are of the following sources and were used at the indicated dilutions: rabbit anti-Iba1 (1:1000, Wako, 019-19741), Chicken anti-GFAP (1:1000, Abcam, ab4674), rabbit anti-Olig2 (1:200, Abcam, ab109186), mouse anti-βIII Tubulin (1:300, Abcam, ab78078), rabbit anti-CD45 (1:500, Abcam, ab10558), rabbit anti-NeuN (1:300, Abcam, ab177487). Secondary antibodies conjugated to Alexa-488, Alexa-594, or Alexa-647 were from abcam and diluted 1/500. Sections were incubated with primary antibody in a humidified chamber overnight at 4 °C, rinsed three times in PBS, incubated with secondary antibody for 2 h at room temperature, rinsed three times in PBS, incubated with DAPI (1/200 in PBS, Solarbio, C0060), rinsed twice in PBS. Finally, coverslips were mounted. For BDA labeling, Alexa Fluor® 594 streptavidin conjugate (1/200 in PBS, Invitrogen, S32356) was added to the secondary antibody solution and incubated with secondary antibodies.

### Quantitative analyses

Quantification of stained tissue sections was performed by Image Pro Plus (IPP) software. For the quantification of microglia (Iba1+), astrocytes (GFAP+), oligodendrocytes (Olig2+), the total number of immunolabeled cells per cross section was counted at ×10 magnification immunofluorescence images obtained using a microscope equipped with a DP71 camera. For the quantification of neurons (βIII Tubulin^+^), the number of immunolabeled cells in 400 × 300 μm sectors from spinal cord ventral horn was counted at ×20 magnification immunofluorescence images. For the quantification of cell body size of astrocytes or microglia, the longest diameter of cell body of one astrocyte or microglia per cross section was measured at ×20 magnification images. For the quantification of process lengths of astrocytes, the longest process of one astrocyte per cross section was measured at ×20 magnification images. The length of longest process was the linear distance from the initial part to farthest end of the process. For the quantification of lesion area, lesion site was manually outlined according to anti-GFAP immunofluorescence and quantified by IPP software. For the quantification of fluorescence intensity of CD45+ cells surrounding the lesion, Mean gray values of CD45 staining is determined in three randomly selected 150 × 150 μm sectors around the lesion and the average represent the mean fluorescence intensity per sample. For the quantification of axonal dieback distance, the vertical distance from the farthest end of BDA+ process to vertical line of spinal cord long axis, which pass through the lesion epicenter was measured at ×10 magnification images.

### In vivo T2-weighted magnetic resonance imaging (T2W-MRI)

T2W-MRI acquisitions were done at 3 weeks after injury using a small animal-designed 7.0 T MRI scanner (Bruker, PharmaScan; Bruker BioSpin, Germany) with a 38-mm quadrature volume coil was used. Mice were anesthetized (1.5% isoflurane) using a MR-compatible anesthetic equipment and T2 sequences were conducted using the Bruker ParaVision 5.0 system. After that, mice were placed back warm cages.

### Behavioral analysis

Recovery of locomotor function after SCI was quantified in an open field using the Basso Mouse Scale (BMS), according to the method developed by Basso and colleagues^16^. The BMS is a 10-point locomotor rating scale, in which 9 point means normal locomotion while 0 equals to complete paralysis. Two investigators, who are blinded to the experiment, independently gave the score of the mice.

### Statistics

Statistical significances were evaluated using Student’s *t-*test, one-way or two-way ANOVA. Two-tailed unpaired Student *t*-test was used to determine the significance of differences between two groups. One-way ANOVA followed by Tukey post hoc test was used for comparisons of three or more groups. Two-way ANOVA followed by Bonferroni post-tests was used for multiple comparisons. All statistical analyses were performed using the GraphPad Prism software version 5.0 (GraphPad Software Inc.). Values were presented as mean±standard deviation(SD). A *p*-value < 0.05 was considered as statistically significant.

## Results

### Efficient depletion of microglia in the adult spinal cord

**Fig. 1:**
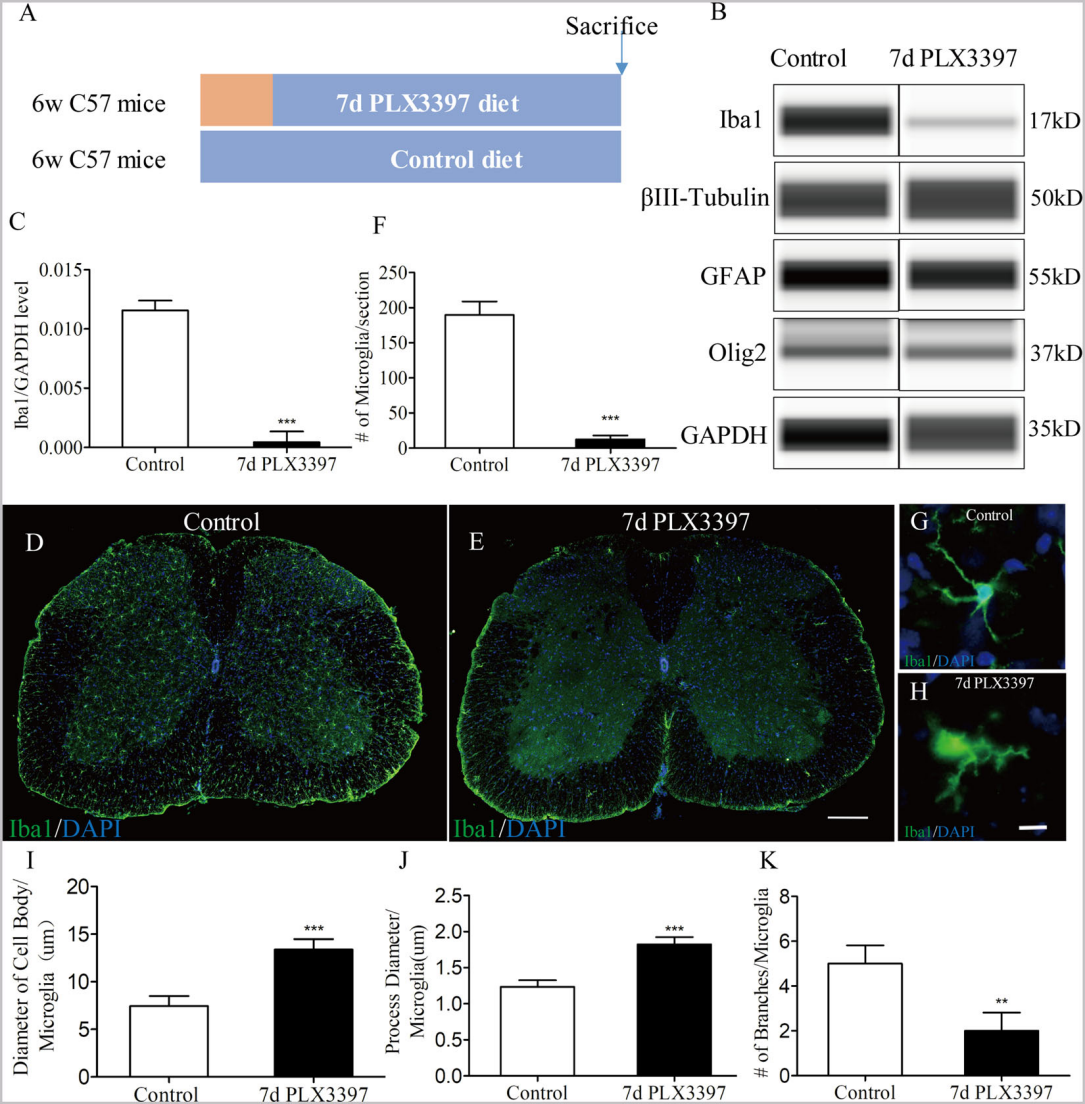
Efficient depletion of microglia in the adult spinal cord via pharmacologic inhibition of CSF1R. **a** Schematic of the experimental design: 6-week-old C57BL/6 mice were fed either PLX3397 or control chow for 7 days. On day 7, mice were euthanized for western blots or Immunostaining. **b** Western blot analysis of spinal cord homogenates for steady state levels of the microglia marker Iba1, neuronal markers βIII Tubulin, the oligodendrocyte marker Olig2, and the astrocyte markers GFAP. **c** Quantification of Iba1 in (B) showing CSF1R inhibition decreased Iba1 protein levels (*n* = 4 per group). **d**, **e** Representative immunofluorescence images of mouse spinal cord sections showing Iba1^+^ ramified microglia (green). Almost no Iba1^+^ cells were detected in mice that were fed a PLX3397 diet for 7d. **f** Quantification of the number of IBA1^+^ cell in the spinal cord from control and PLX3397-treated mice (n = 4 per group) as shown in (D) and (E). **g**, **h** Iba1 immunostaining shows changes in microglia morphology with representative microglia shown from control and 7-day PLX3397-treated mice. I-K Microglial morphology were assessed by the diameter of cell body per microglia (**i**), the process diameter per microglia (**j**) and the number of branches per microglia (**k**) (*n* = 4 per group). Data are expressed as mean ± SD. Scale bars: **d**, **e**, in **e** 200 µm; **g**, **h**, in **h** 10 µm.

Diet-mediated application of the CSF1R inhibitor PLX3397 was shown previously to deplete microglia efficiently in mouse adult brains and optic nerves^5,12^. As spinal cords were not analyzed in these studies, we first addressed this aspect by processing these spinal cords from mice on normal or PLX3397 diet for the expression of Iba1, a marker for microglia. Spinal cords were homogenized, and western blots were performed. As expected, mice with 7-day PLX3397 treatment were found to have decreased levels of Iba1. The Iba1 protein levels decreased to 95% below the levels of the control diet group (Fig. 1b, c). Immunostaining for Iba1 in the spinal cord of these animals confirmed the results and further revealed a clear decrease in microglia numbers with PLX3397 treatments (Fig. 1d–f). Only single remaining cells were very occasionally found with a larger cell body, an increased thickness of processes, and a reduction in the number of branches (Fig. 1g–k), typically associated with a more phagocytotic phenotype^17^. These data indicate that PLX3397 can be used to selectively and nearly completely eliminate spinal cord microglia under steady state in vivo conditions in mice.

### Microglial depletion does not affect other cell types in spinal cord

**Fig. 2:**
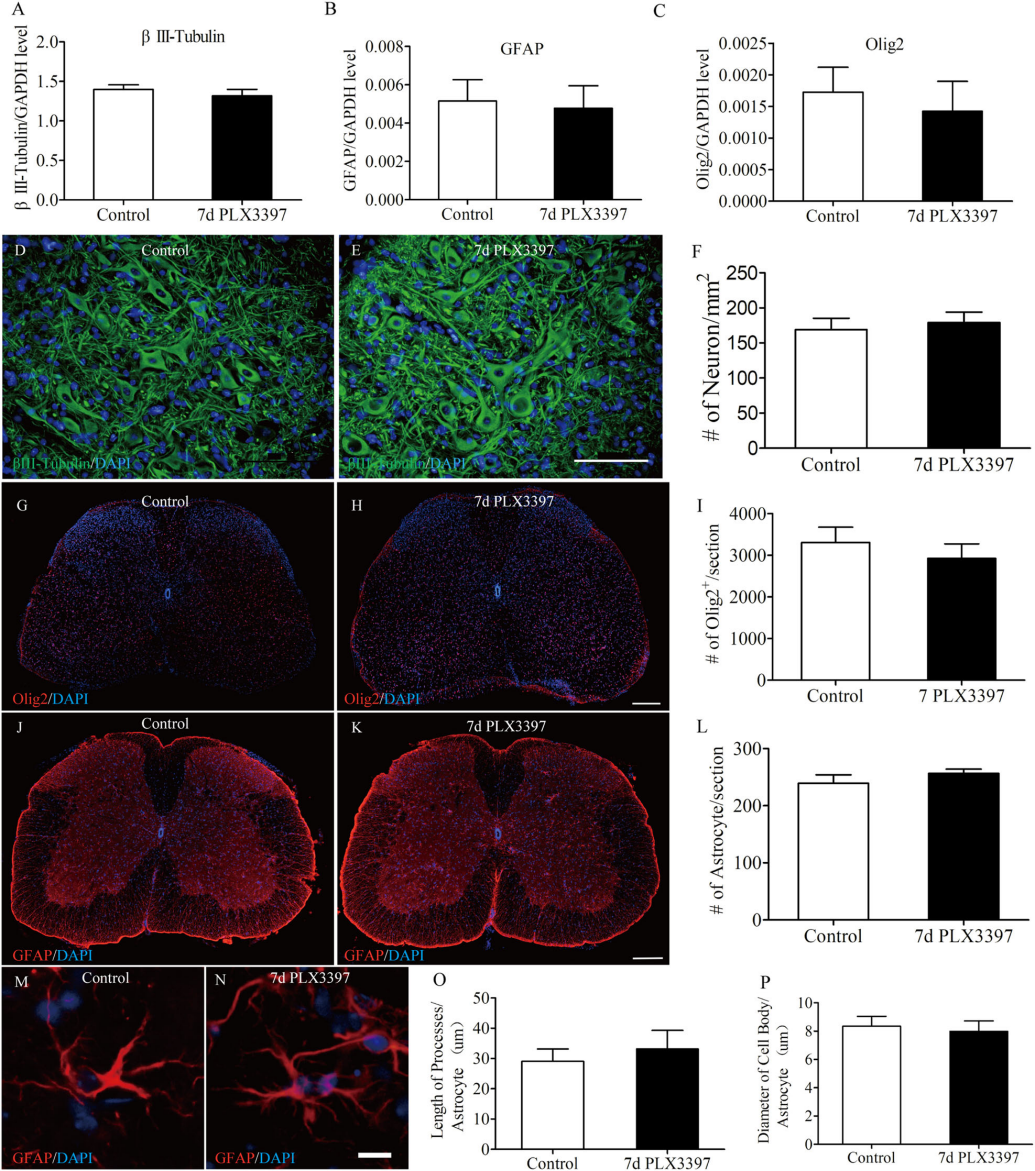
Microglia elimination does not affect other cell types in spinal cord. Six-week-old wild-type C57BL/6 mice were treated with PLX3397 or vehicle or 7 days to eliminate microglia. Western blots were performed on spinal cord homogenates for the neuronal markers βIII Tubulin, the oligodendrocyte marker Olig2, and the astrocyte markers GFAP (Fig. 1b). **a**–**c** Quantification of βIII Tubulin, Olig2, and GFAP in Fig. 1b shows no significant changes in the expression of the three markers between two groups (*n* = 4 per group). **d**, **e** Representative images taken from mouse spinal cord ventral horn immunostained for βIII-Tubulin (green) and DAPI (blue). **f** Quantification of the number of βIII-Tubulin^+^ cells in the spinal cord from control and PLX3397-treated mice (*n* = 4 per group) as shown in **d** and **e**. **g**, **h** Representative immunofluorescence images of mouse spinal cord sections showing Olig2^+^ cells. **i** Quantification of the number of Olig2^+^ cells in the spinal cord from control and PLX3397-treated mice (*n* = 4 per group) as shown in **g** and **h**. **j**, **k** Representative immunofluorescence images of mouse spinal cord sections (the same sections with Fig. 1d, e, respectively) showing GFAP^+^ cells. **l** Quantification of the number of GFAP^+^ cells in the spinal cord from control and PLX3397-treated mice (*n* = 4 per group) as shown in **j** and **k**. **m**, **n** GFAP immunostaining shows the morphology of astrocytes from control and 7-day PLX3397-treated mice. **o**, **p** Astrocyte morphology were assessed by the length of processes per astrocyte and the diameter of cell body per astrocyte (*n* = 4 per group). Data are expressed as mean ± SD. Scale bars: **d**, **e** in **e** 100 µm; **g**, **h** in **h** 200 µm; **j**, **k** in **k** 200 µm; **m**, **n** in **n** 10 µm.

To explore whether microglial depletion could affect other cell types in spinal cord, we test protein expression levels of neuronal, oligodendrocytic, and astroctytic markers via western blot (Fig. 1b) No changes in markers βIII Tubulin, oligodendrocyte transcription factor (Olig2), or glial fibrillary acidic protein (GFAP) were observed (Fig. 2a–c). Immunofluorescent stains for βIII Tubulin, Olig2 or GFAP in the spinal cord of these mice confirmed the results. βIII Tubulin+, Olig2+ or GFAP + cell counts showed no differences with microglial depletion (Fig. 2d–l). Given the important crosstalk between astrocytes and microglia, we further performed morphological analyses of astrocytes with 7 or 14 days of PLX3397 treatment. The results revealed that no changes in astrocyte cell body sizes or process lengths after microglial depletion (Fig. 2m–p and Supplementary Fig. 1). Therefore, elimination of microglia results in no changes in cell numbers or morphology.

### CSF1R blockade reduce microglial accumulation induced by SCI

**Fig. 3:**
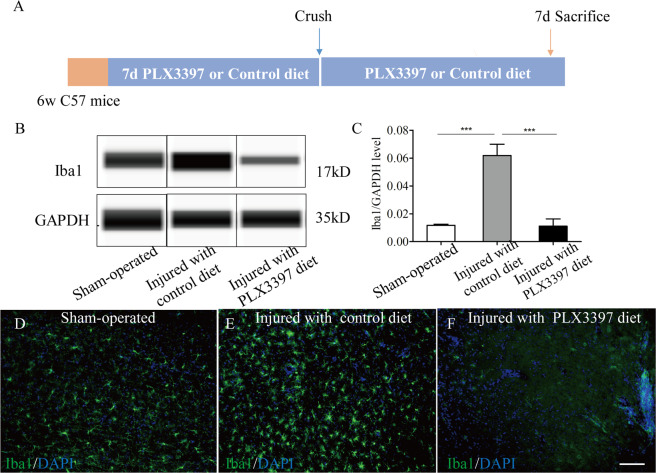
CSF1R inhibition reduces microglial accumulation induced by SCI. **a** Schematics of experimental design showing the timeline of microglia depletion, spinal cord crush injury, and sacrifice. **b** Western blot analysis of spinal cord homogenates from sham-operated, injured with control or PLX3397-treated mice (*n* = 4 per group) for steady state levels of the microglia marker Iba1. **c** Quantification of **b** showing a robust increase in levels of Iba1 expression at 7 days following SCI. CSF1R inhibition reverses the increase induced by SCI. **d**–**f** Representative immunofluorescence images of mouse spinal cord sections showing Iba1^+^ microglia. The changes in number of Iba1^+^ microglia consist with the changes of Iba1 expression levels. Data are expressed as mean ± SD. Scale bars: **e**, **f** in **f** 100 µm.

To explore the effect of microglia depletion in SCI context, wild-type C57BL/6 mice were fed with PLX3397 or control diet for 7 days prior to sham-operation (only performed laminectomy) or SCI procedures (Fig. 3a). Thereafter, these mice continued to receive PLX3397 or control diet until 7 days post injury (dpi). The spinal cords of these mice were isolated form spinal columns for immunofluorescent stains or western blot. Western blot analysis of spinal cord homogenates showed a robust increase in levels of Iba1 expression at 7 days after full crush injury. Treatment with CSF1R inhibition prevented this SCI-induced Iba1 increase (Fig. 3b, c). Immunofluorescent stains for Iba1 in the spinal cord of mice fed with control diet showed that microglia accumulated around the lesion site at 7 dpi and exhibited a round morphology, which points to a potential increase in their phagocytic activity. In the spinal cord of mice with PLX3397 treatment, microglia around the lesion epicenter dramatically reduce at 7 days following SCI (Fig. 3d–f). Thus, our data indicate that microglia are rapidly recruited around the site of SCI and CSF1R blockade reduce this accumulation induced by SCI.

### Microglial depletion reduces locomotor recovery after SCI

**Fig. 4:**
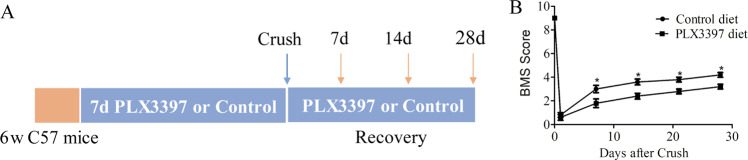
Microglial depletion reduces locomotor recovery after SCI. **a** Schematics of experimental design showing the timeline of microglia depletion, spinal cord crush injury, behavioral testing using the BMS score. **b** Locomotor function was assessed using the BMS score over a 28-day period after SCI (*n* = 4 per group). Data are expressed as mean ± SD. *p < 0.05.

We next investigated the role of microglia in functional recovery after SCI. Chow containing either PLX3397 or no drug was given to C57BL/6 mice starting 7 days prior to SCI and then maintained for an additional 4 weeks (Fig. 4a). The effect of PLX3397-mediated CSF1R inhibition and reduced microglial accumulation on motor behavior following SCI was evaluated using BMS scores. We showed that mice depleted of microglia exhibited impaired locomotor recovery compared to mice treated with control diet at 7, 14, 21, and 28 dpi (Fig. 4b). Thus, the results suggest that microglia play an essential role in motor function recovery after SCI.

### Microglial depletion disorganizes astrocytic scar formation

**Fig. 5:**
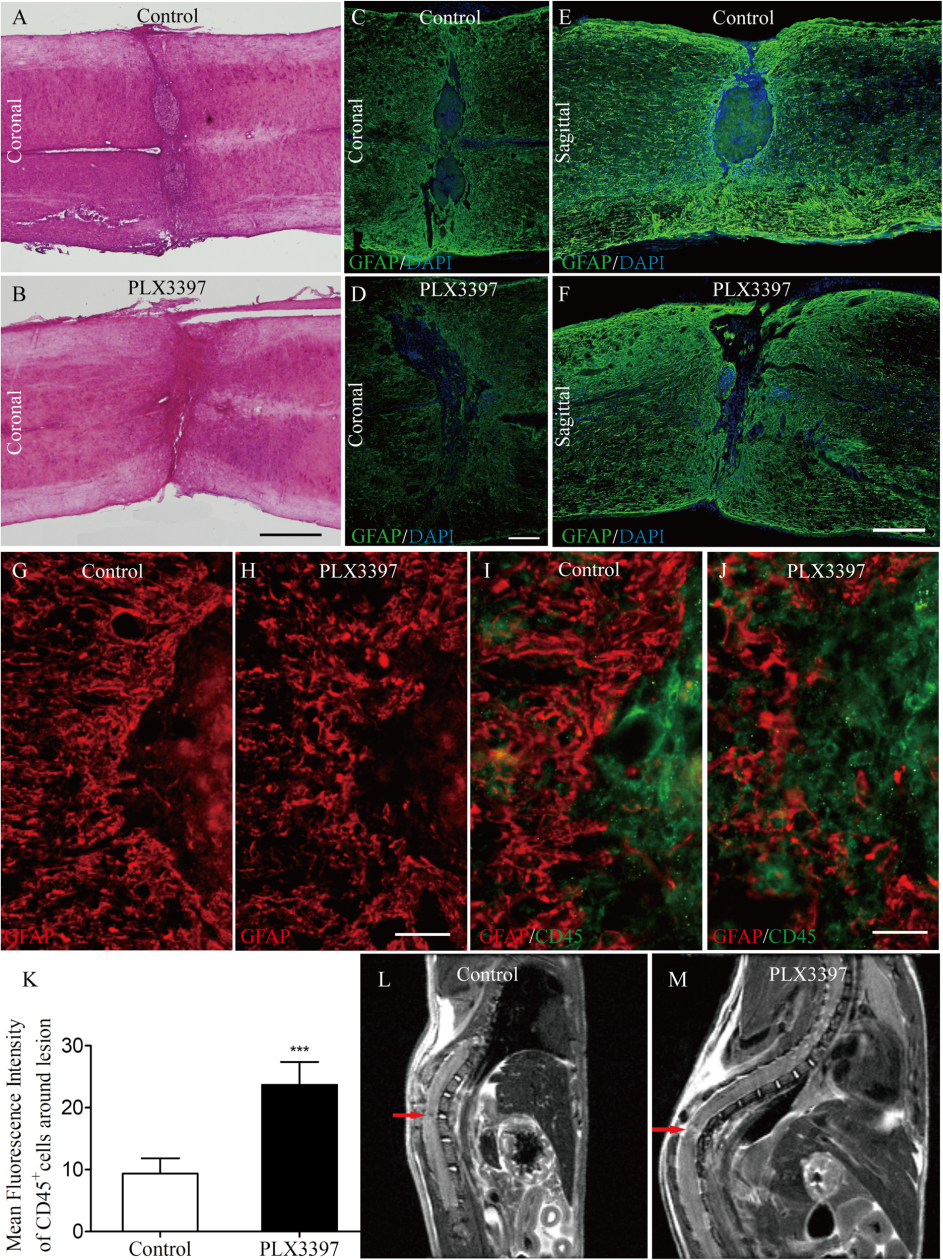
The elimination of microglia results in a disorganized astrocytic scar at the lesion border. **a**–**f** Representative HE (**a**, **b**) or immunofluorescence (**c**–**f**) images of coronal (**a**–**d**) or sagittal (**e**, **f**) sections of mouse spinal cord taken at 30 dpi. In control mice (**a**, **c**, **e**), astrocytes adjacent to the lesion form a compact scar. This astrocytic scar was compromised in mice depleted of microglia using PLX3397 (**b**, **d**, **f**). **g**–**j** Representative immunofluorescence images of spinal cord sections taken at the lesion epicenter at 21 dpi. In mice fed with the control diet (**g**, **i**), astrocytes exhibit elongated processes oriented parallel to the lesion border. This orientation was disorganized in PLX3397-treated mice (**h**, **j**) and associated with clusters of CD45^+^ immune cells spreading outside of the lesion epicenter. **k** Quantification of the infiltration degree of CD45+ cells spreading outside of the lesion epicenter in the spinal cord from control and PLX3397-treated mice (*n* = 4 per group) as shown in **i** and **j**. **l**, **m** In vivo T2W-MRI assessment of the lesion in control and PLX3397-treated mice after SCI. In vivo sagittal sections of mouse spinal cord taken at 21 dpi. Red arrows represent abnormal signals in the spinal cord. Scale bars: **a**, **b**, in **b** 1 mm; **c**, **d**, in **d** 200 µm; **e**, **f**, in **f** 200 µm; **g**, **h**, in **h** 50 µm; **i**, **j**, in **j** 25 µm.

In cases of injury to the spinal cord, a key pathological event is the formation of a glial scar induced by reactive astrogliosis^18^. Whether scar formation contributes to functional recovery following CNS injury remains a matter of debate. Recently, Anderson and his colleagues^19^ showed that astrocyte scar formation aids CNS axon regeneration. Thus, we next explored whether microglia play an important role in the formation of the astrocytic scar that develops following SCI. Strikingly, both HE and Immunofluorescence stain showed astrocytes around the lesion core formed a less compact scar when microglia were depleted using PLX3397 compared to the control treatment at 30 dpi (Fig. 5a–f). Notably, we observed that GFAP + astrocytes were oriented randomly and not aligned in any particular direction in PLX3397-treated SCI mice at 21 dpi (Fig. 5g, h). This disorganized astroglial scar was accompanied by an increased infiltration of CD45^+^ cells into the spinal cord parenchyma (Fig. 5i–k), which may lead to an increase of intramedullary high-signals in SCI mice with PLX3397 treatment (Fig. 5l, m).

### Microglial depletion delays the repopulation of the lesion site by astrocytes after SCI

**Fig. 6:**
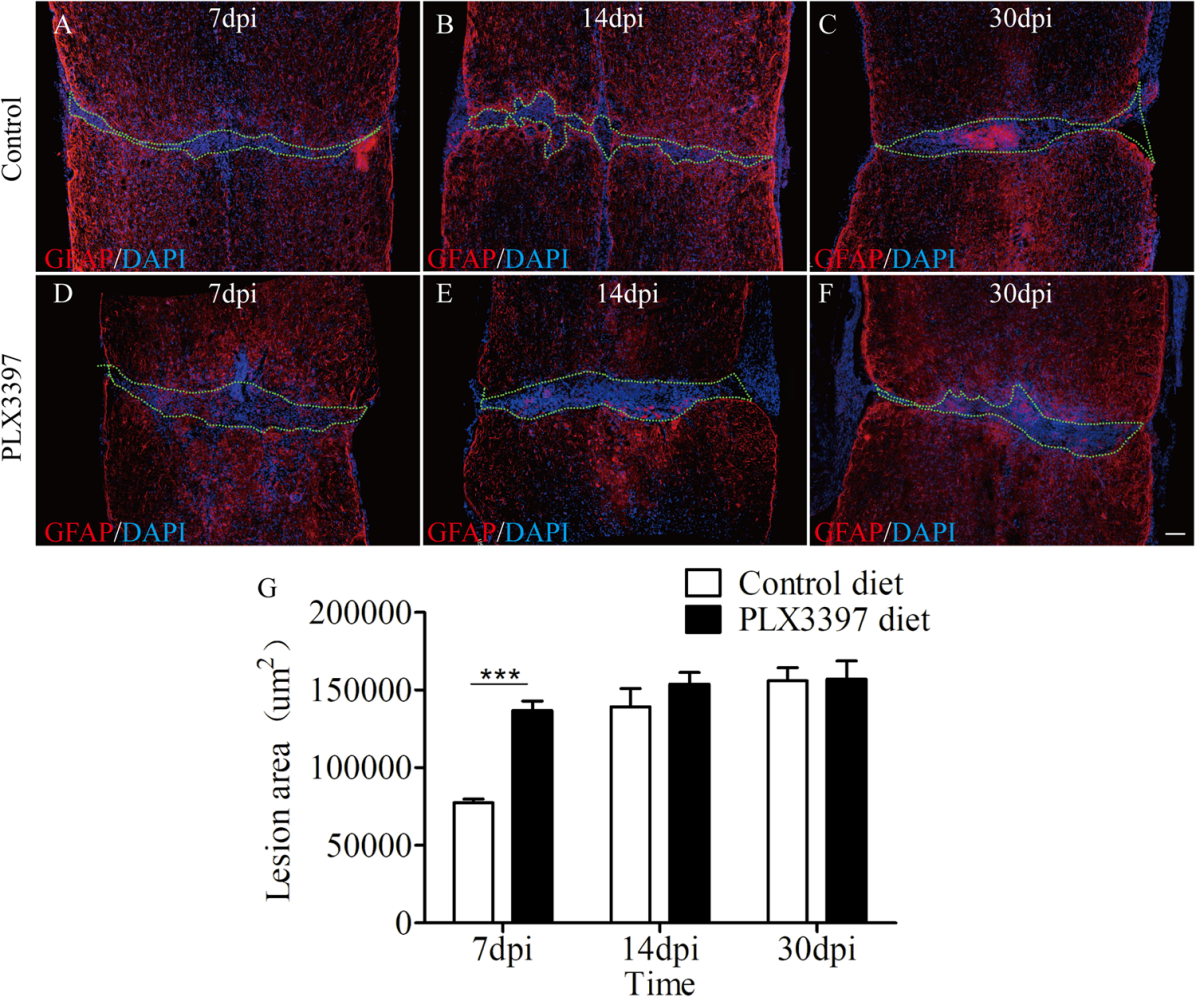
Delayed repopulation of the spinal cord lesion site by astrocytes upon microglia depletion. **a**–**f** Representative photographs of coronal sections of mouse spinal cord taken from mice on either control diet or PLX3397at 7, 14, and 30 dpi. The area circled by green dashed line represent lesion area. **g** Quantification of the lesion size of the spinal cord from control and PLX3397-treated mice (*n* = 4 per group) as shown in **a**–**f**. Data are expressed as mean ± SD. **p < 0.01. Scale bars: **a**–**f**, in **f** 100 µm.

It was proposed that reactive astrocytes exert protective functions after SCI^20^. We next investigated whether microglial depletion affects tissue damage. Immunofluorescence staining for GFAP revealed that the lesion area significantly increased in PLX3397-treated mice compared to that in control treated mice at 7 days post SCI, because a larger, seemingly cell-free area with strongly reduced GFAP staining was observed in these PLX3397-treated spinal cord (Fig. 6a, d). Quantification revealed that microglial depletion was correlated with an increase of the lesion core area at 7 dpi. However, no significant differences in lesion area between groups were detected at 14 and 30 dpi, although lesion sizes tended to be slightly larger in PLX3397-treated mice (Fig. 6g). Thus, microglia seem to have a rather transient role in this process and their absence just delayed astrocyte repopulation of the lesion site.

### Microglial depletion reduces neuronal survival and exacerbates axonal dieback

**Fig. 7:**
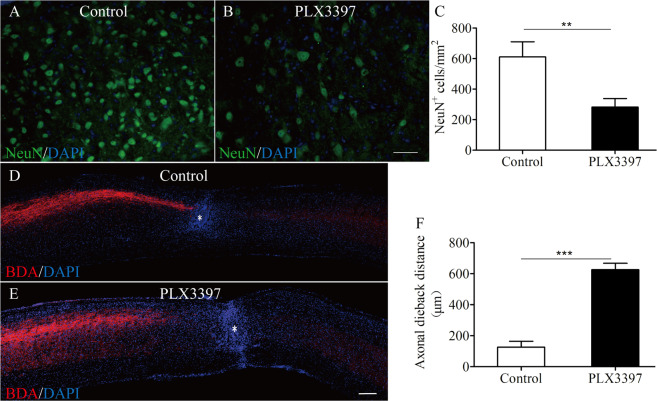
Microglial depletion reduces neuronal survival and exacerbates axonal dieback. **a**, **b** Representative images taken around the lesion epicenter at 30 dpi immunostained for NeuN (green) and DAPI (blue). **c** Quantification of the number of NeuN^+^ cells in the spinal cord from control and PLX3397-treated mice (*n* = 4 per group) as shown in **a** and **b**. **d**, **e** Representative sagittal sections of the spinal cord from control and PLX3397-treated mice at 8 weeks after injury. BDA-labeled CST axons are shown in red. Blue represents DAPI immunostaining. White asterisk represents lesion epicenter. **f** Quantification of axonal dieback distance at 8 weeks post SCI as shown in **d** and **e** (*n* = 4 per group). Data are expressed as mean ± SD. **p < 0.01, ***p < 0.001. Scale bars: **a**, **b**, in **b** 50 µm; **d**, **e**, in **e** 200 µm.

Having established that the lesion area was increased at 7 dpi and functional recovery worse at 7, 14, 21, and 28 dpi in microglia-depleted mice, we next investigated whether microglial depletion would influence neuronal survival. Immunofluorescence staining for NeuN revealed that there were fewer neurons around lesion epicenter in spinal cord sections of PLX3397 diet group compared with control diet groups at 30 dpi (Fig. 7a–c). Given microglial depletion delays the repopulation of the lesion site by astrocytes after SCI, and previous study that astrocytes might bridge a lesion site to support axonal regeneration^19,21^, we next analyzed the impact of microglia depletion on axon regeneration in vivo. We performed a full crush SCI at T10 in mice fed with either PLX3397 diet or control diet, injected the axon tracer BDA into the right cortex 6 weeks later, and perfused these mice 8 weeks after SCI. Sagittal sections through the lesion site were stained for BDA to visualize CST axons. DAPI immunoreactivity was used to visualize cellular nuclei and the lesion. Data revealed that axonal dieback distance in PLX3397-treated mice was significantly increased than that in control treated mice at 8 weeks post SCI (Fig. 7d–f). Altogether, our results indicate that microglia play a neuroprotective role during SCI.

## Discussion

The role of microglia in SCI is still ambiguous because both beneficial and detrimental effects have been proposed. Here, we took advantage of newly developed pharmacological depletion strategies that allow us to target microglia specifically to investigate their role in mouse SCI. We demonstrated that PLX3397 efficiently and specifically depleted spinal microglia without any discernable effect on other cell populations in spinal cord. In the context of traumatic SCI, PLX3397 also could efficiently reduce microglial accumulation induced by SCI. Notably, the near-complete microglial elimination by PLX3397 treatment led to the disruption of glial scar formation, which was accompanied by an increased infiltration of blood-derived inflammatory cells into the spinal cord parenchyma. Upon a full crush injury, microglia depletion delayed the repopulation of the spinal lesion site by astrocytes, reduced neuronal survival, exacerbated axonal dieback, and finally impaired locomotor function recovery compared to controls.

Previous studies demonstrated that oral administration of CSF1R inhibitor efficiently resulted in elimination of ~90% of all microglia brain-wide^8,12,22^. This newly developed depletion strategies were more efficient than a genetic approach, by which diphtheria toxin has to be injected into transgenic mice to induce cell death^23^. Treatment with CSF1R inhibitor also avoided the occurrence of the undesired cytokine storm, a massive increase in cytokine and chemokine production in genetic model^24^. As observed in these studies on brain, we found that continuous treatment with the CSF1R inhibitor PLX3397 also resulted in the depletion of spinal cord microglia (93%), because almost no Iba1+ cells were detected in spinal cord. Additionally, other cell types in spinal cord, such as neurons, astrocytes, and oligodendrocyte also remained unaffected upon PLX3397 treatment. Notably, treatment times might possibly be reduced from 3 weeks to 1 week, because we found that 1 week of PLX3397 diet was sufficient to achieve near-complete depletion, similar to previous studies in brain or optic nerves^5,12^.

SCI triggers a robust inflammatory response that includes the rapid activation and migration of microglia around the lesion site. The current study consists with this process and Iba1+ cells were also not detectable in spinal cord of injured mice fed with PLX3397 diet. Thus, PLX3397 is similarly effective in the uninjured and injured spinal cord. All these make pharmacological treatments with CSF1R inhibitor become a better method to investigate the specific role of microglia in SCI.

Microglial activation occurs very early after CNS injury. Because of the multiple potentially conflicting capabilities of activated microglia, their overall impact on CNS recovery remains debatable^2^. Traditionally, activated microglia are generally thought to be detrimental to CNS recovery following injury, because the release of proinflammatory cytokines might convey neurotoxicity to injured and healthy neurons^25,26^. Upregulation of neurotoxic genes in microglia was detected as early as 1 day post SCI^27^. Liddelow and his colleagues^5^ showed that activated microglia secrete interleukin (IL)-1α, tumor necrosis factor alpha (TNFα), and C1q, and these cytokines together induce neurotoxic reactive astrocytes, which lose many normal astrocyte functions and induce death of neurons. Accordingly, administration of neutralizing antibodies to IL-1α or TNFα reduces neuronal cell death and improves functional recovery after SCI^28–30^. However, the current study standing in contrast to previous discoveries uncovered a rather beneficial impact of activated microglia on SCI because depletion of microglia disrupted glial scar formation, reduced neuronal survival, delayed astrocyte repopulation, exacerbated axonal dieback, and impaired locomotor recovery. Consistent with our results, growing studies reported beneficial effects of microglia in CNS injury^8,31–34^. Especially, one recent study demonstrated that elimination of microglia via administration of the dual CSF1R/c-Kit inhibitor PLX3397 exacerbates postischemic inflammation and brain injury^31^. This again demonstrated that the overall effect of activated microglia in CNS injury is beneficial.

As the most abundant cell type in the spinal cord, astrocytes provide trophic support for neurons, promote synapse formation, maintain homeostasis^35,36^. Astrocytes respond to CNS injury through a process called reactive astrogliosis, and form a glial scar with other cell types, including microglia^36^. Functions of reactive astrocytes have been a matter of some debate, with previous studies showing they are both helpful or harmful to CNS recovery^19,36,37^. Here, we found that microglia regulate the astrocytic response. In the absence of microglia, glial scar formation was perturbed. In line with our results is a previous study, where suppression of microglial proliferation by the cell cycle inhibitor olomoucine attenuated astroglial scar formation after SCI^38^. This disorganized astroglial scar promotes spread of leukocyte outside of the lesion core, which was accompanied by a reduced neuronal survival, worsened axonal retraction, and attenuated motor recovery. In addition, the repopulation of lesion site by reactive astrocytes was markedly delayed upon microglia depletion. Increased spread of inflammatory cells had also been reported before following selective and conditional ablation of reactive astrocytes after SCI^20^, as well as in mice with conditional deletion of scar-forming gene from astrocytes^39,40^. Given growing evidence on beneficial roles of reactive astrocytes, we speculate that microglia may provide neuroprotection by modulating astrocyte response after SCI.

However, current data cannot conclude that regulation of the astrocyte response is the only way that contributes to the neuroprotection of microglia after CNS injury. Microglia may play neuroprotection via directly modulating neuronal activity after SCI. As previously mentioned, microglia reduced excitotoxic neuronal injury by regulating intracellular calcium levels^8^, but how microglia could regulate the neuronal activity remains unclear. Jin et al. suggested that conditioned media of microglia could reduce ischemia-induced neural injury, suggesting the possibility that microglia-derived factors may be beneficial for ischemic neurons. Whether this direct interaction between microglia and neurons also exist in context of SCI will certainly need to be investigated in future studies.

In conclusion, our data reveal a neuroprotective role of microglia after SCI. The protection may result from their modulating on the formation of the astroglial scar, and thus sequester blood-derived inflammatory cells in the lesion core so that avoiding inflammation-mediated tissue damage.

## Supplementary information

Supplementary figure 1

Supplementary information
