# Supplementary figures and images for: Depletion of microglia exacerbates injury and impairs function recovery after spinal cord injury in mice
Source: Cell Death Dis. 2020 Jul 13;11(7):528. doi: 10.1038/s41419-020-2733-4 (PMC7359318; doi:10.1038/s41419-020-2733-4)

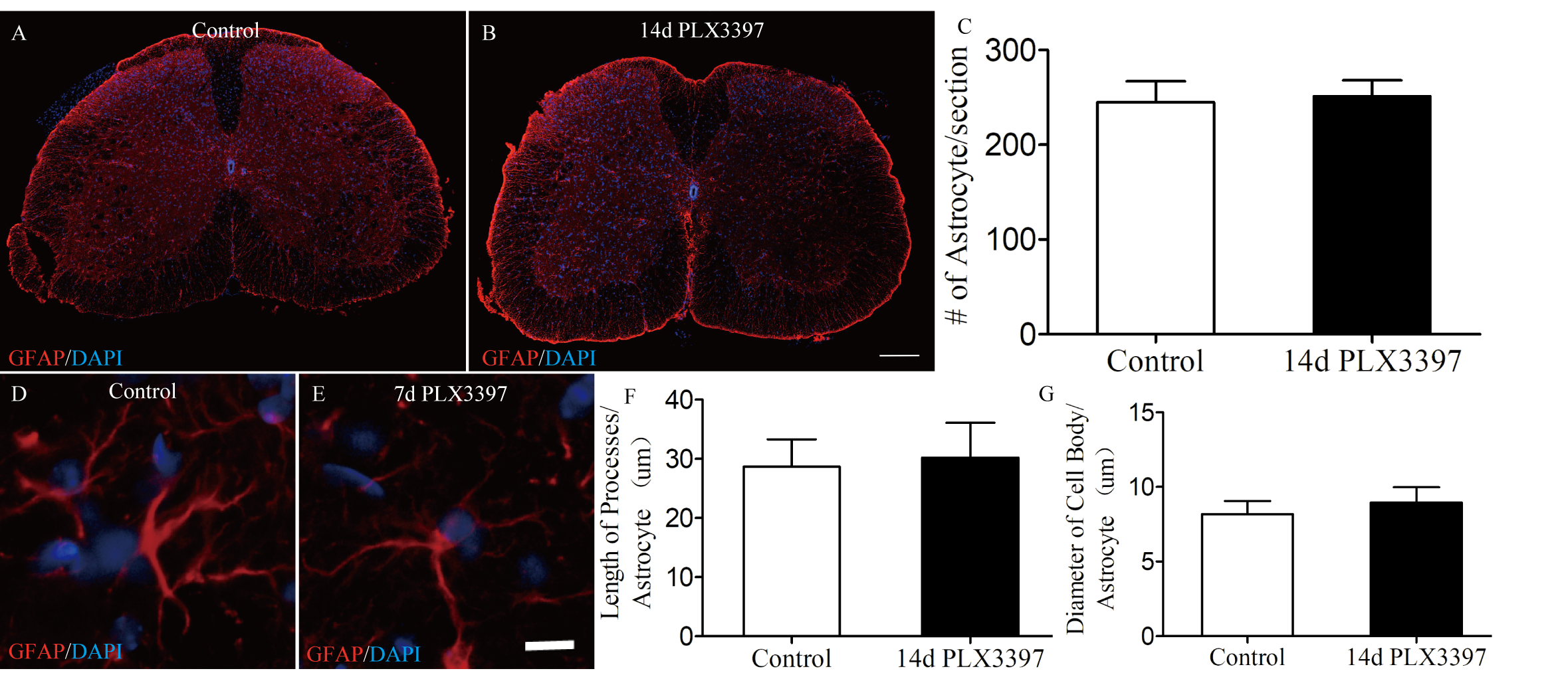

Supplement: Supplementary file 1 — Supplementary figure 1 [file 41419_2020_2733_MOESM1_ESM.tif]
