# Supplementary material for: Depletion of microglia exacerbates injury and impairs function recovery after spinal cord injury in mice
Source: Cell Death Dis. 2020 Jul 13;11(7):528. doi: 10.1038/s41419-020-2733-4 (PMC7359318; doi:10.1038/s41419-020-2733-4)
Supplement: Supplementary file 2 — Supplementary information [file 41419_2020_2733_MOESM2_ESM.docx]

Supplemental Fig.1 14 days of microglia elimination does not affect astrocyte response. A,B Representative immunofluorescence images of mouse spinal cord sections showing GFAP+ cells. C Quantification of the number of GFAP+ cells in the spinal cord from control and 14-day PLX3397-treated mice (n = 4 per group) as shown in (A) and (B). D,E GFAP immunostaining shows the morphology of astrocytes from control and 14-day PLX3397 treated mice. F,G Astrocyte morphology were assessed by the length of processes per astrocyte and the diameter of cell body per astrocyte (n = 4 per group). Data are expressed as mean ± SD. Scale bars: (A,B in B) 200 µm; (D,E, in E) 10µm
